# An investigation of Y-TZP surface characteristics and adhesive properties following treatment with Tri-biochemical Silica Coating, Femtosecond Laser, and Nano-hydroxyapatite: A scanning electron microscopy evaluation

**DOI:** 10.12669/pjms.41.3.11302

**Published:** 2025-03

**Authors:** Fahad Alkhudhairy, Nourah N. Shono

**Affiliations:** 1 Fahad Alkhudhairy Restorative Dental Sciences Department, College of Dentistry, King Saud University, Riyadh, Saudi Arabia; 2 Nourah N. Shono Restorative Dental Sciences Department, College of Dentistry, King Saud University, Riyadh, Saudi Arabia

**Keywords:** Dentistry, Mechanical testing, Surface Modification, Surface characteristics, Shear bond strength, Zirconia Polycrystals

## Abstract

**Objectives:**

To examine how advanced surface pretreatment techniques - Tribochemical silica coating (TBC), Femtosecond laser (FS), and Nano-hydroxyapatite (HA) coating - affect the surface roughness (Ra) and shear bond strength (SBS) between resin luting cement and Yttria-stabilized tetragonal zirconia polycrystals (Y-TZP)

**Methods:**

The lab-based comparative study was approved by the ethical committee of King Saud University under IRB number F98-971X9 and was completed in three months (June to September, 2024). Eighty-four YTZP zirconia discs were created and sorted into four groups based on different conditioning agents: Group-I (APA) as the control, Group-II (TBC), Group-III (FS laser), and Group-IV (Nano-HA coating). Ra was measured on five discs from each group using a profilometer, while surface topography was examined through SEM analysis. Ten specimens per group were bonded with luting cement, followed by SBS testing and fracture mode analysis using a universal testing machine and stereomicroscope, respectively. One-way ANOVA with post hoc Tukey tests (p<0.05) was used for statistical analysis of Ra and SBS means across the groups.

**Results:**

Group-IV (Nano-HA coating) exhibited the highest Ra and SBS values. Conversely, Group II (TBC) displayed the lowest Ra and weakest bond strength.

**Conclusion:**

Nano-HA coating and FS laser can be employed as viable alternatives to APA without compromising the physical and mechanical properties of zirconia ceramic.

## INTRODUCTION

Yttria-stabilized tetragonal zirconia polycrystals (Y-TZP) are highly regarded in indirect restorations for their excellent mechanical and aesthetic properties.^1^ Zirconia’s unique transformation toughening sets it apart from other dental ceramics. However, achieving a strong bond with resin-luting cement remains challenging.^2^ Extensive research has focused on modifying zirconia’s surface chemistry to improve its shear bond strength (SBS) with adhesive cement.^3^ Air-particle abrasion (APA), or sandblasting, is the leading method for enhancing surface roughness (Ra) and SBS between luting cement and zirconia.^4^ It is important to note that grit blasting causes a phase transformation from tetragonal to monoclinic structure, potentially weakening Y-TZP and increasing fracture risk.^5^

Tribochemical silica coating (TBC) is an advanced air-blasting technique for applying silica to zirconia surfaces. It uses silica-modified aluminum oxide (Al_2_O_3_) particles to clean the surface and increase Ra, which enhances micromechanical retention and chemical bonding.^6^ However, indexed literature provides inconclusive results on TBC’s effects on Ra and resin cement adhesion to zirconia, indicating a need for further research.^7^ Laser technology in dentistry improves the mechanical properties of teeth and dental materials via surface modification.^8^ The Femtosecond laser (FS) is notable for micromachining surfaces, like zirconia ceramics, without thermal damage.^9^ This technology ablates materials in thin layers while preserving their properties.^10^ Nonetheless, comprehensive data on its feasibility for conditioning zirconia in dental reconstruction is lacking.

In prosthodontics, nanotechnology is emerging as a promising technique. Notably, Nano-hydroxyapatite (HA) serves as an effective ceramic surface modifier, with crystal sizes ranging from 50 to 1000 nm.^11^ Studies indicate that nano-HA forms a thin, uniform coating on ceramics, providing sufficient strength. A laboratory study by Atri et al. investigated Nano-HA for zirconia surface modification, showing shear bond strength (SBS) comparable to air particle abrasion (APA).^12^ However, research in this domain is still limited, requiring further investigation. The present study assumed that the Ra of Y-TZP treated with TBC, FS laser, and Nano-HA coating would be comparable to APA. It also anticipated that the SBS results for zirconia modified with these techniques would align with the control group. Thus, the research aimed to assess the effectiveness of surface pretreatment agents on the Ra and adhesion strength of Y-TZP to resin-luting cement.

## METHODS

The lab-based comparative study followed a checklist for reporting in vitro study (CRIS) guidelines. The study was completed in three months (June to Sep 2024). The study employed 84 YTZP zirconia discs (IPS e.max ZirCAD), measuring 4 × 4 × 4 mm, cut with a slow-speed saw (Isomet Buehler, Lake Bluff, IL, USA). The discs were ultrasonically cleaned in 96% ethanol for 10 minutes and air-dried. The specimens were divided into four groups (n=16 per group) based on the conditioning agent used. Group-I (APA), the control group, underwent air-particle abrasion with 50 μm Al_2_O_3_ particles (Mega OX, Megablast, Brazil) using a sandblasting apparatus (Talleres Mestraitua, Espana) at 2.5 bar pressure, 10 mm distance, for 15 seconds, followed by ultrasonic cleaning. Group-II (TBC) zirconia plates were treated with silica-modified aluminum oxide (Rocatec Soft sand, 3M) at 2 bars of pressure for 15 seconds, with the tip placed 5 cm from the surface, followed by ultrasonic cleaning. Group-III (FS laser) specimens were treated with an FS laser (Coherent Quantronix-Integra-C system, Hamden, CT, USA) using parameters of 800 nm wavelength, 200 mW output power, and 1 kHz repetition rate for 12 minutes. Group-IV (Nano-HA coating) involved preparing a slurry with 10 g HANPs powder (Merck, Germany, <100 nm particles) and 50 cc distilled water, incorporating 1 g polyvinyl alcohol (Merck, Germany) as a binder. The mixture was heated on a magnetic stirrer at 1000 rpm and 100°C for one minute for uniformity. The zirconia blocks were immersed in the slurry at a 45° angle for five seconds.

### Ethical statement:

The research experiments conducted in this article were approved by the Ethical Committee of King Saud University under IRB number F98-971X9, dated June 21, 2024.

### Surface Roughness evaluation:

Ra values were measured using a Contour GT profilometer (Bruker, CA, USA). Three readings were taken from the central 2 mm area of each disc, and the average Ra for each specimen was calculated from these readings.^13^

### Bonding of luting cement:

Clearfil Ceramic Primer Plus was applied to 44 samples using an applicator. A Teflon mold (2 × 2 mm) was placed perpendicular to the disc surface and filled with Panavia V5, a resin cement from Kuraray Noritake Dental (Tainai, Niigata, Japan), mixed according to the manufacturer’s instructions. The cement was photo-polymerized for 60 seconds using a New Light VL-II LED curing unit (GC, Tokyo, Japan). The specimens were then immersed in deionized water and stored at 37°C for a week.

### Thermal aging of samples:

Replicating one year of clinical use was conducted with a thermocycler (Mechatronik, Feldkirchen-Westerham, Germany) in 10,000 cycles. Each cycle involved immersing discs in 5°C and 55°C water baths for 30 seconds each, with a five-second transition between baths.

### SBS assessment:

Bond strength was evaluated using a universal testing machine (UTM) (Autograph AG-X 20kN, Shimadzu, Kyoto, Japan). A perpendicular force was applied to the resin cement-zirconia interface at a constant rate of 0.5 mm/min until separation. The debonding force was measured in Megapascals.

### Surface topography assessment using SEM and Failure mode assessment:

The morphology of surface-conditioned and resin-cement-bonded zirconia plates was evaluated using scanning electron microscopy (LEO-1530VP; GmbH, Oberkochen, Germany). Gold sputter-coated plates were examined at 20 kV with 100× magnification. Failure modes were classified as adhesive, cohesive, or admixed using a stereomicroscope (Carl Zeiss Jena GmbH, Göttingen, Germany) at 40× magnification.

### Statistical analysis:

Statistical analyses were performed using SPSS 28.0 (SPSS, Chicago, IL, USA). The normality of data was assessed using the Kolmogorov-Smirnov test. One-way ANOVA and subsequent Tukey post hoc tests compared Ra and SBS mean across test groups. A p-value below 0.05 indicated statistical significance.

## RESULTS

### SEM evaluation:

**Fig.1:**
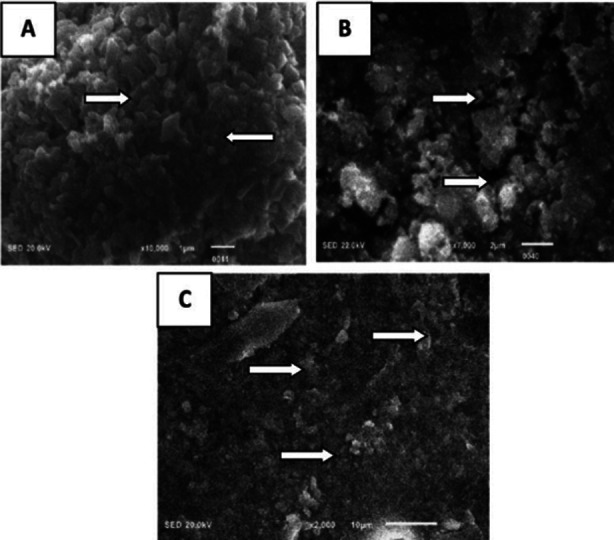
(A) Y-TZP coated with Nano-HA exhibited a surface pattern reminiscent of rose petals, as revealed by SEM analysis (arrows). (B) Topographical examination of Y-TZP modified by FS-laser showed uniform and well-defined surface patterns, with evidence of microcrack melting and crystal fusion (arrow). (C) The SEM image of Y-TZP with TBC silica coating illustrated the melting and bonding process of silica onto the zirconia surface (arrow).

In Fig.1 microscopic examination of Y-TZP Coatings (A) Scanning electron microscopy shows Y-TZP coated with Nano-HA, exhibiting a rose petal-like morphology (indicated by arrows). (B) Topographical assessment reveals a highly organized Nano-HA surface with consistent patterns horizontally and cross-sectionally, and signs of microcracks or crystal melting (arrow highlights this) when Y-TZP treated with FS-L. (C) SEM imaging of Y-TZP with a tribo-chemical silica coating shows a clear contrast to the Nano-HA coating, with visible evidence of silica melting and bonding to the zirconia substrate (arrow points to this).

### Ra analysis:

Surface roughness (Ra) measurements on Y-TZP after different pretreatments. Nano-HA treatment (Group-IV) showed the highest Ra values, while TBC (Group-II) resulted in the smoothest surfaces represent in Table-I. Statistical analysis indicated no significant difference between FS laser (Group-III) and Nano-HA treatments (p > 0.05). The APA technique (Group-I) produced intermediate roughness, with Ra values higher than TBC but lower than FS laser and Nano-HA (p < 0.05).

**Table-I:** Ra on Yttria-stabilized tetragonal zirconia polycrystals following surface modifications.

Investigated groups	Mean ± SD (µm)
Group-I: APA	1174.11±0.051*
Group-II: TBC	1033.57±0.028^**^
Group-III: FS laser	1430.15±0.069^***^
Group-IV: Nano—HA coating	1441.47±0.062^***^

ANOVA: Air-particle abrasion (APA), tribo-chemical silica coating (TBC), Femtosecond laser (FS), Nano-Hydroxyapatite (HA). Different numbers of

* denote statistically significant differences! (Post Hoc Tukey)

### Bond strength analysis:

Table-II presents the shear bond strength (SBS) of Y-TZP to resin luting cement after various pretreatments. Group-IV (Nano-HA) had the highest bond integrity, while Group-II (TBC) had the lowest SBS. No significant difference was found between Group-III (FS laser) and Group-IV (p > 0.05). Group-I (APA) displayed SBS values higher than Group-II but lower than Groups 3 and 4 (p < 0.05).

**Table-II:** SBS of Yttria-stabilized tetragonal zirconia polycrystals to resin luting cement after using different surface modification regimes.

Experimental groups	Mean ± SD (MPa)	p- value!
Group-I: APA	14.08±0.31*	<0.05
Group-II: TBC	12.78±0.16^**^	
Group-III: FS laser	17.81±0.42^***^	
Group-IV: Nano-HA coating	18.61±0.56^***^	

ANOVA: Air-particle abrasion (APA), tribo-chemical silica coating (TBC), Femtosecond laser (FS), Hydroxyapatite (HA). Different numbers of

* denote statistically significant differences! (Post Hoc Tukey.

### Bond failure mode assessment:

**Fig.2:**
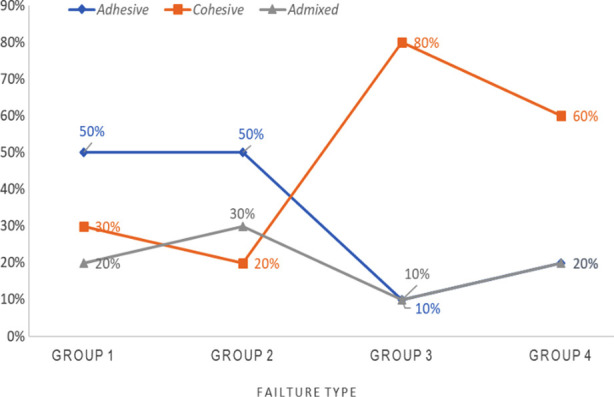
Modes of failure distribution in different experimental groups.

Fig.2 The distribution of failure modes in the experimental groups is shown. Cohesive failure was most common in Groups 3 and 4, whereas Groups 1 and 2 exhibited diverse failure types.

## DISCUSSION

The study hypothesized that Y-TZP’s Ra and SBS would be comparable to APA when treated with TBC, FS laser, and Nano-HA coating. However, results showed significantly different Ra and SBS values for all experimental groups compared to APA. The SBS test was used, acknowledging its limitations, as alternative specimen preparation techniques could introduce interfacial defects. Crack propagation during tensile loading might cause early interface failure at reduced stress levels.^14^ Attia et al. state that the acceptable bonding range in restorative dentistry is 10-13 MPa. The use of nano-HA coating with FS laser treatment significantly increased Ra values and bond strength.^15^ Various methods, including thermal coating, can enhance zirconia ceramics, as shown by Azari et al.^16^

The study demonstrated that Nano-HA coating improves SBS by forming a thin, uniform, and strong layer. This coating also increases Ra on implant surfaces, aligning with previous research findings.^16^ The increase in Ra values is due to the SEM analysis showing a rose petal-like morphology on Y-TZP discs. Conversely, Moezzizadeh et al. found that Nano-HA coating yielded lower bond strength than sandblasting for zirconium samples.^17^ The superior Ra and bond integrity observed with FS laser are due to its ability to engrave a cross-stripe pattern on the Y-TZP surface, enhancing micro-retention of the resin luting agent and improving SBS.^18^ FS laser machining is highly effective in ceramic microprocessing due to its ultra-brief pulse duration and high peak power, enabling localized melting, vaporization, or ablation during a very short laser-material interaction period.^19^ SEM analysis of FS-conditioned zirconia ceramic discs supports these findings, revealing well-organized, consistent, and delineated patterns without visible microcracks. Laboratory studies by Yeğin et al., Akpinar et al., and Prieto et al. reported similar results.^18^,^20^–^22^

The literature review indicates that while APA roughens zirconia surfaces for better bonding, it can weaken the material’s mechanical properties. However, Blatz et al. found that using APA with 50 μm alumina particles at pressures of 2.5 bar or less reduces damage while creating sufficient microporosity and wettability, thereby improving bond strength.^23^ Thus, this study used air-abrasion on specimens with 50 μm Al_2_O_3_ particles at 2.5 bar pressure, with increased Ra values confirmed by SEM analysis. Conversely, discs pretreated with TBC showed significantly lower Ra and SBS scores compared to the control group. Research shows that silica coating helps absorb kinetic energy during impact, leading to minor modifications and lower Ra scores.^24^ Nagaoka et al. suggested that silica-coated alumina particles impact the zirconia surface, converting some kinetic energy to thermal energy and causing a localized temperature rise.^25^ This results in partial melting of some silica particles, which then bond to the zirconia surface. Simultaneously, some alumina particles fracture and some silica particles remain unmelted, depositing on the surface. These non-fused silica particles negatively affect the bond strength of luting cement.^25^ However, studies have shown that using TBC with silane enhances the zirconia surface’s chemical interaction susceptibility. The present study highlights the alternate methods to improve surface characteristics and adhesive strength of Y-TZP to improve clinical outcomes and treatment prognosis in patients.

### Limitations:

The study’s methodology had inherent limitations. Conducted in a lab, the findings may not directly apply to clinical situations. The investigation focused on individual FS laser parameters’ effects on Ra and bond strength, and the bond strength assessment was limited to one type of resin cement. The authors suggest future research to examine HA coating and FS laser effects on zirconia’s Ra and SBS in clinical settings. Also, the effect on other mechanical properties following surface modifications needs to be evaluated.

## CONCLUSION

Zirconia ceramic’s physical and mechanical properties remain stable with nano-hydroxyapatite coating or Femtosecond laser techniques, providing alternatives to air particle abrasion.

### Authors’ Contribution:

**FA and NNS:** Data collection, study design, manuscript writing, manuscript drafting, data analysis, final manuscript approval and are accountable for the integrity of the study.
